# Unique occurrence of trichilemmal carcinoma in the scrotal skin of a giant panda: a pathological analysis

**DOI:** 10.1186/s12917-026-05668-5

**Published:** 2026-06-26

**Authors:** Chengyao Li, Zongliang Xiong, Shanshan Ling, Yan Zhu, Linhua Deng, Mengfang Yang, Chengdong Wang, Qian Wang, Danyu Chen, Mao Huang, Kao Wang, Desheng Li, Zhengli Chen, Caiwu Li

**Affiliations:** 1 Key Laboratory of SFGA on the Giant panda, China Conservation and Research Center for the Giant Panda, Chengdu, China; 2 Experimental Animal Disease Model Research Laboratory, School of Veterinary Medicine, Sichuan Agricultural University, Chengdu, China; 3 Dujiangyan People’s Hospital, Chengdu, China

**Keywords:** Giant panda (Ailuropoda melanoleuca), Trichilemmal carcinoma, Histopathology, Immunohistochemistry, Surgical treatment

## Abstract

**Background:**

Trichilemmal carcinoma (TC) is a rare cutaneous appendage malignancy with scarce human cases and very few animal cases have been documented in the available literature, especially in giant pandas.

**Case presentation:**

A 23-year-old male captive giant panda had 4-week scrotal hyperplasia and ulceration, with a 4.5 cm×4 cm irregular lesion and neutrophils were elevated. Pathology revealed dermal lobular/cord-like invasive atypical clear cells, squamous nests and outer root sheath cysts. PAS staining was positive. Immunohistochemistry showed Her-2 (+), CK5/6 (++), p63 (++), Ki-67 (+), CD34 (+), and p53 (+), excluding squamous cell carcinoma and trichofolliculoma. Local resection (0.8 cm margin) was done under general anesthesia. Postoperative anti-infection led to 2-week wound healing; 3-month follow-up showed no recurrence with normal behavior.

**Conclusions:**

This first reported giant panda TC with human-like immunophenotype provides evidence for managing rare animal cutaneous appendage tumors.

## Background

Trichilemmal carcinoma (TC) is a relatively rare malignant tumor of skin appendages, originating from the outer root sheath cells of hair follicles. Currently, there is no clear global incidence data available, and relevant statistics are mostly based on clinical case reports. However, its incidence is much lower than that of common cutaneous malignant tumors, usually accounting for less than 1%. To date, there have been no reported cases or relevant research studies on trichilemmal carcinoma in animals. In humans, trichilemmal carcinoma mostly occurs in hair - dense areas such as the scalp, and occasionally in the face, trunk, extremities and other parts. In the early stage, it usually presents as an isolated, slowly growing nodule or plaque with a relatively hard texture, smooth or rough surface, and the color is mostly skin - colored, light red or brown. As the disease progresses, ulceration, bleeding and crusting may occur in the center, forming an ulcer with raised and irregular edges. The skin lesions of some patients may show cauliflower - like hyperplasia.

Regarding the etiology of trichilemmal carcinoma, it is currently generally believed to be related to factors such as ultraviolet radiation, chronic inflammatory stimulation, genetic factors and low immune function. Malignant proliferating trichilemmal tumors, which are characterized by invasive growth, prominent trichilemmal keratinization and marked nuclear pleomorphism on hematoxylin and eosin (H&E) staining, have been found to harbor several genetic abnormalities. Studies have identified such alterations in these tumors, including TP53 mutations and amplification of ERBB2 (HER2), BRD4 and TYMS genes [1, 2]. Miki Nishio et al. found that the expression of tumor suppressor genes such as MOB1 was significantly reduced in patients with trichilemmal carcinoma, and 100% of mice with partial MOB1 deletion developed cutaneous outer root sheath carcinoma, which may be one of the main causes of trichilemmal carcinoma [3].

Histopathological examination is the gold standard for the diagnosis of trichilemmal carcinoma. The key diagnostic criteria include lobular or nest-like growth patterns centered on pilosebaceous units, predominance of atypical clear cells with abundant glycogen‑rich cytoplasm, invasive “pushing” growth into the dermis, and trichilemmal keratinization characterized by peripheral palisading of tumor cells with abrupt central keratinization and absence of a granular layer [4]. The clear cytoplasm contains glycogen, showing a positive Periodic Acid - Schiff (PAS) reaction, which is sensitive to amylase. Additional histopathological features include fibrous tissue proliferation, vasodilation around tumor cells, and occasionally a distinct transparent zone at the periphery of tumor lobules. Immunohistochemically, the tumor expresses high - molecular - weight keratin, while carcinoembryonic antigen (CEA) and epithelial membrane antigen (EMA) are usually negative. These morphological and immunohistochemical characteristics are critical for distinguishing trichilemmal carcinoma from its differential diagnoses, particularly squamous cell carcinoma and trichofolliculoma. Immunohistochemistry plays an increasingly important auxiliary role: for instance, CD34 positivity helps differentiate trichilemmal carcinoma from squamous cell carcinoma (which is usually CD34-negative), while overexpression of p53 and a high Ki − 67 proliferation index confirms the malignant nature of the tumor.

In terms of treatment, local surgical resection [5] or micrographic Mohs surgery with a sufficient tumor - free margin [6] is the recommended treatment for trichilemmal carcinoma. For postoperative wound repair, new techniques such as reader flaps can be used, which can achieve tension - free suture of large circular skin defects with minimal scarring and minimal resection of additional healthy skin.

## Case presentation

**Fig. 1:**
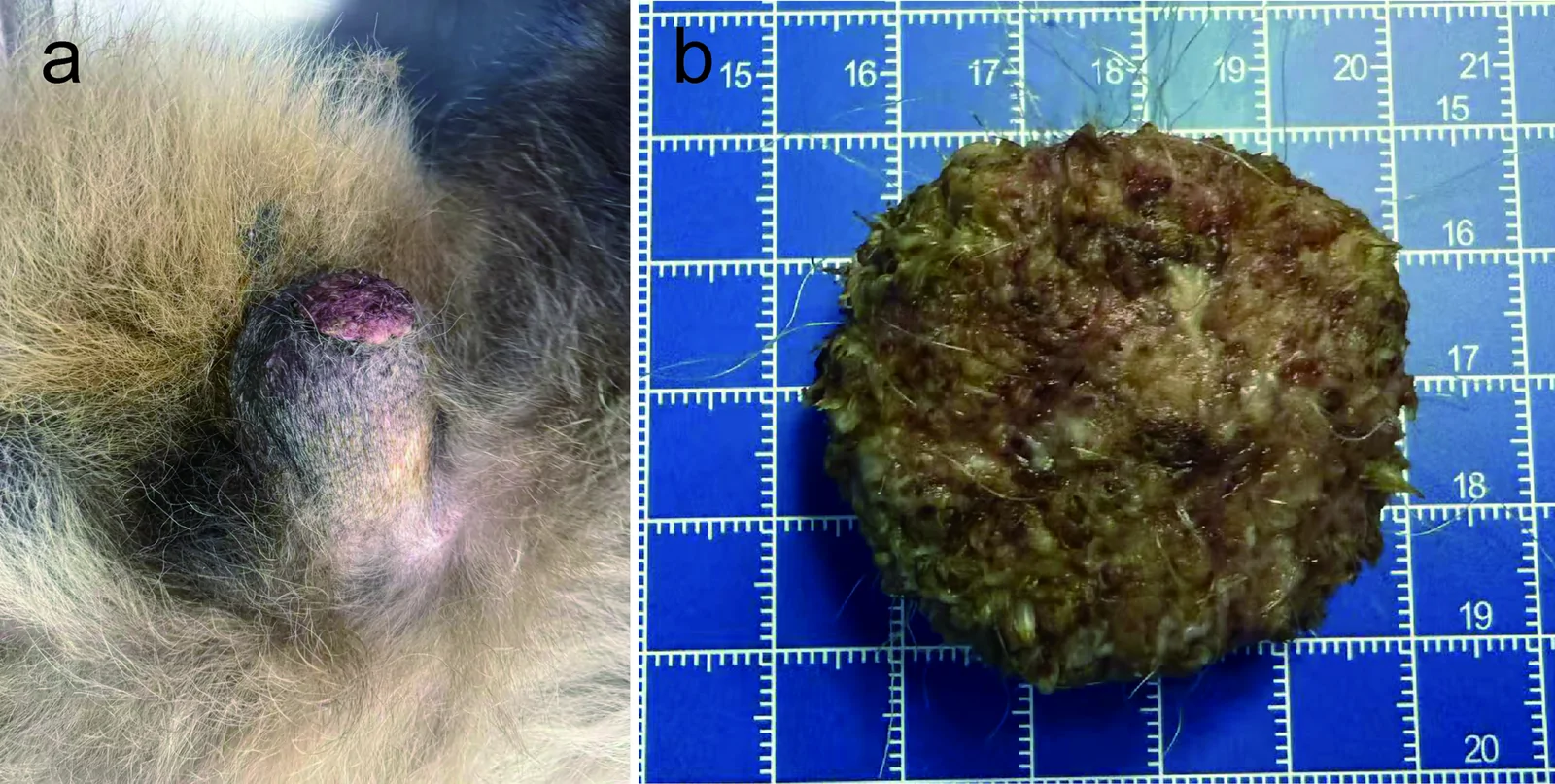
A scrotal skin tumor in a giant panda.** a** A tumor located on the skin of the right scrotum. **b** The removed tumor. Size : 4.5 cm×4 cm

We report a case of a 23-year-old male giant panda that underwent surgical resection. A lesion on the right scrotal skin was first noticed by the breeders, who promptly reported the abnormality to the veterinary team. The veterinary staff initiated continuous monitoring of the lesion, during which it was observed that the lesion showed significant rapid growth over a 15-day period (Fig. 1a). Close clinical examination revealed an ulcer proliferative growth measuring 4 cm × 4.5 cm (Fig. 1b), with visible areas of hemorrhage and necrosis on the surface of the lesion. No evidence of regional lymphadenopathy was observed during the physical examination. A wide local excision was performed under general anesthesia, and the resected tissue sample was submitted for histopathological examination to confirm the diagnosis of trichilemmal carcinoma. Blood samples were also collected for laboratory tests.

After completing preoperative laboratory tests to confirm the absence of surgical contraindications, the veterinary team administered general anesthesia to this 23-year-old male giant panda (body weight: 100 kg), a mandatory step given that giant pandas are large, ferocious wild animals. The anesthesia regimen started with an intramuscular injection of tiletamine hydrochloride and zolazepam hydrochloride for injection (Zoletil^®^ 100, Virbac China, Beijing) at a dosage of 3 mg/kg, followed by endotracheal intubation and the use of a veterinary anesthesia machine (WATO EX-35Vet, Shenzhen Mindray Animal Medical Technology Co., Ltd., Shenzhen) to maintain anesthesia with isoflurane (Shenzhen RWD Life Science Co., Ltd., Shenzhen). During the surgery, the “tumor-free operation” principle was strictly followed: the lesion was resected locally, with a 0.8 cm margin of normal skin tissue preserved at the excision edge to ensure thoroughness.

Postoperatively, targeted nursing measures were implemented. On one hand, antimicrobial and anti-inflammatory treatments were administered: povidone-iodine (Haishi Hainuo Group Co., Ltd) was applied topically for disinfection twice daily, mupirocin ointment (GlaxoSmithKline (Tianjin) Co., Ltd) was applied topically twice daily, and 1 g of cefalexin capsule (Sunflower Pharmaceutical Group Co., Ltd) was given three times daily for three consecutive days. The surgical site was closely monitored for abnormal signs such as redness, exudation, or necrosis. On the other hand, the panda’s activity range was restricted to prevent the incision from splitting due to friction or traction.

Following two weeks of meticulous care, the surgical wound healed completely, with full epidermal coverage and no signs of infection or poor healing. Postoperative follow-up revealed no induration, mass formation, or local recurrence at the wound site. Additionally, the panda’s food intake and activity frequency returned to preoperative levels, with no abnormal behaviors or fluctuations in physiological indicators observed. These findings further confirm the effectiveness of the surgical treatment and the panda’s excellent postoperative recovery.

Laboratory tests show an increase in the number of neutrophils, with no significant abnormalities in coagulation function, which meets the surgical criteria (Tables 1 and 2). In addition, we also found that the indicators such as globulin (GLB), glutathione reductase (GR), serum type IV collagen (IVC), alkaline phosphatase (ALP), α-1-fucosidase (AFU), and lactate dehydrogenase (LDH) all increased to varying degrees.

**Table 1:** Biochemical test report of giant panda

Test	Results	Unit	Reference	Evaluation
α-AMY	1579.9	U/L	751.67-1524.85 [22]	high
β2MG	0.34	mg/L	0-0.91 [22]	-
ALT	81	U/L	61.92–101.6 [22]	-
AST	81	U/L	58.59–85.27 [22]	-
AST/ALT	1		0.73–1.09 [22]	-
TP	66.8	g/L	61.95–70.17 [22]	-
ALB	21.6	g/L	28.15–32.97 [22]	low
GLB	45.2	g/L	31.31–39.67 [22]	high
A/G	0.48		0.74–1.02 [22]	low
TBIL	1.0	µmol/L	1.18–2.46 [22]	low
DBIL	0.19	µmol/L	0.07–0.57 [22]	-
IBIL	0.81	µmol/L	0.86–2.14 [22]	-
GGT	15	U/L	4.28–16.8 [22]	-
ALP	538	U/L	78.47-155.67 [22]	high
TBA	33.9	µmol/L	12.67–68.01 [22]	-
PA	8	Mg/L	7.18–24.9 [22]	-
AFU	6	U/L	3.56–5.72 [22]	high
ADA	24.1	U/L	4.09–7.3 [22]	high
5’-NT	0.73	U/L	1.18–3.3 [22]	low
CHE	1074	U/L	969.64-1323.44 [22]	-
Urea	21.67	mmol/L	3.22–6.32 [22]	high
CREA	245.55	µmol/L	83.19-153.31 [22]	high
UA	49	µmol/L	18.68–48.6 [22]	-
GLU	4.18	mmol/L	3.09–4.55 [22]	-
TC	4.72	mmol/L	3.79–7.37 [22]	-
TG	2.66	mmol/L	1.67–2.61 [22]	-
HDL	2.91	mmol/L	2.49–3.43 [22]	-
LDL	1.67	mmol/L	2.15–4.67 [22]	low
ApoA1	1.18	g/L	0.53–0.89 [22]	high
ApoB	0.02	g/L	0.01–0.07 [22]	-
K	4.39	mmol/L	4.57–5.49 [22]	low
Na	120	mmol/L	120.86-127.22 [22]	-
Cl	87.9	mmol/L	91.3-96.76 [22]	low
Ca	1.11	mmol/L	1.96–2.18 [22]	low
LDH	955.00	U/L	302.83-943.39 [22]	high
CK	129.00	U/L	149.19-240.73 [23]	low
CKMB	< 0.10	ng/mL	193.27-328.39 [23]	low
HBDH	642.00	U/L	250.8-845.8 [23]	-
PT	13.0	Sec	19.7–31.9(*n* = 128)	Low
APTT	30.2	Sec	21.7–48.6(*n* = 128)	-
Fog	2.24	g/L	1.7–3.35(*n* = 128)	-
TT	18.2	Sec	12.7–24.3(*n* = 128)	-
CA724	14.47	U/ mL	2.88–15.29(*n* = 10)	-
FT3	3.3	pmol/L	0.58–5.9 [24]	-
FT4	15.62	pmol/L	1.71–10.22 [24]	high
T3	1.14	nmol/L	0.25–0.96 [24]	high
TSH	< 0.01	mIU/L	4.16–20.66 [24]	low
TgAb	1.25	IU/mL	12.06–17.27 [24]	low
NO	27	µmol/L	3–60(homo)	-
GR	257	U/L	33–73(homo)	high
INR	1.18		0.85–1.25(homo)	-
BNP	< 10	pg/mL	< 100(homo)	-
cTnI	84.76	pg/mL	<=18(homo)	high
IVC	110.22	ng/ml	0–90(homo)	high
MYO	3.63	ng/mL	16.5–95.2(homo)	low
HCY	26.9	µmol/L	<=15(homo)	high
T4	18.78	nmol/L	61.13-164.74(homo)	low

The Beckman AU5800 (Beckman Coulter, Inc. USA) is used for biochemical test. Some of the indicators are derived from the published reference standards for giant pandas, while others use the human reference range

*α-AMY* amylase, *β2MG* β2-Microglobulin, *ALT* Alanine Aminotransferase, *AST* Aspartate Aminotransferase, *TP* Total Protein, *ALB* Albumin, *GLB* Globulin, *TBIL* Total Bilirubin, *DBIL* Direct Bilirubin, *IBIL* Indirect Bilirubin, *GGT* Gamma-Glutamyl Transferase, *ALP* Alkaline phosphatase, *TBA* Total Bile Acids, *PA* Phosphatase Acid, *AFU* Alpha-L-Fucosidase, *ADA* Adenosine Deaminase, *5’-NT* 5’- Nucleosidase, *CHE* Cholinesterase, Urea, Carbamide, *CREA* Creatinine, *UA* Blood uric acid, *GLU* Glucose, *TC* Total Cholesterol, *TG* Triglyceride, *HDL* High-density lipoprotein, *LDL* Low-Density Lipoprotein, *ApoA1* apolipoprotein A-I , *ApoB* Apolipoprotein B, *K* Potassium, *Na* Serum Sodium, *Cl* Chloride, *Ca* Calcium, *LDH* Lactate Dehydrogenase, *CK* Creatine Kinase, *CKMB* creatine kinase isoenzymes, *HBDH* Hydroxybutyrate Dehydrogenase, *PT* Prothrombin time, *APTT* Activated Partial Thromboplastin Time, Fog, Freezing of gait, *TT* Thrombin time, *CA724* Carbohydrate Antigen724, *FT3* Free Triiodothyronine, *FT4* Free Thyroxine, *T3* Total Triiodothyronine, *TSH* Thyroid-Stimulating Hormone, *TgAb* Anti-Thyroglobulin Antibody, *NO* Nitric Oxide, *GR* Glutathione reductase, *INR* International normalized ratio, *BNP* Brain Natriuretic Peptide, *cTnI* Cardiac troponin I, *IVC* Inferior Vena Cava, *MYO* Myoglobin, *HCY* Homocysteine

**Table 2:** Routine blood test report of giant panda

Test	Results	Unit	Reference	Evaluation
WBC	8.78	10^9^/L	6.54–9.22 [23]	-
NEUT%	83.9	%	65.43–72.17 [23]	high
LYMPH%	10.8	%	23.21–30.83 [23]	low
MONO%	5.3	%	3.44–5.74 [23]	-
EO%	0.0	%	0.73–1.55 [23]	low
BASO%	0.0	%	0.02–0.32 [23]	low
NEUT#	7.36	10^9^/L	3.68–6.02 [22]	high
LYMPH#	0.95	10^9^/L	0.74–1.42 [22]	-
MONO#	0.47	10^9^/L	0-0.85 [22]	-
EO#	0.00	10^9^/L	0-0.18 [22]	-
BASO#	0.00	10^9^/L	0-0.03 [22]	-
RBC	5.67	10^12^/L	5.37–6.63 [22]	-
HGB	109	g/L	106.56-126.08 [22]	-
HCT	30.2	%	27.85–33.43 [22]	-
MCV	53.2	fL	49.28–53.28 [22]	-
MCH	19.2	pg	18.73–20.19 [22]	-
MCHC	361	g/L	374.48-385.16 [22]	Low
RDW-SD	28.1	fL	31.67–34.73 [23]	low
RDW-CV	14.9	%	15.43–17.23 [23]	low
PLT	461	10^9^/L	202.83-417.89 [22]	high
PCT	0.227	%	0.26–0.36 [23]	low
MPV	5.7	fL	5.01–7.09 [22]	-
PDW	14.4	fL	5.29–14.57 [25]	-
P-LCR	3.1	%	2.12–3.34 [23]	-

The Mindray BC-760 CS (Mindray Bio-Medical Electronics Co., Ltd., China) is used for routine blood tests

*WBC* Leukocyte, white blood cell, *NEUT% *Neutrophil Percentage, *LYMPH%* Lymphocyte Percentage, *MONO%* Monocyte Percentage, *EO%* Eosinophil Percentage, *BASO%* Basophil Percentage, *NEUT#* Neutrophil Count,* LYMPH#* Lymphocyte Count, *MONO#* Monocyte Count, *EO#* Eosinophil Count, *BASO#* Basophil Count, *RBC* Red Blood Cell, *HGB* Hemoglobin, *HCT* Hematocrit, *MCV* Mean Corpuscular Volume, *MCH* Mean Corpuscular Hemoglobin, *MCHC* Mean Corpuscular Hemoglobin Concentration, *RDW-SD* Red Cell Distribution Width-Standard Deviation, *RDW-CV* Red Cell Distribution Width-Coefficient of Variation, *PLT* Platelet, *PCT* Plateletcrit, *MPV* Mean Platelet Volume, *PDW* Platelet Distribution Width, *P-LCR* Platelet Larger Cell Ratio

**Fig. 2:**
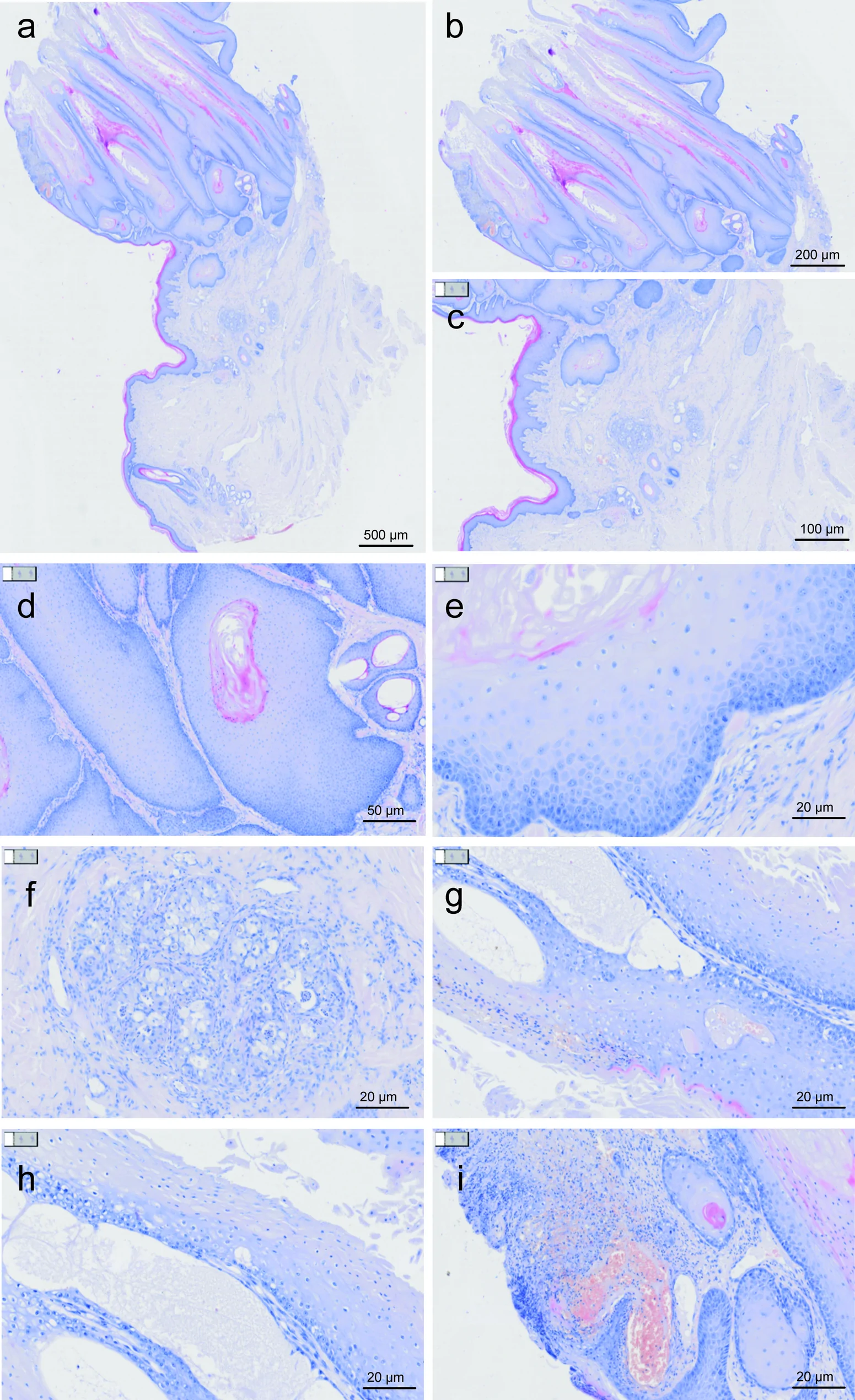
HE staining of tumor tissue sections.** a** Tumor general view, 20X. **b** Tumor general view, 40X. **c** Tumor general view, 100X. **d** Trichilemmal keratinization area, 200X. **e** Trichilemmal keratinization area, 400X. **f** Atypical clear cell area, 400X. **g** Atypical clear cell area, 400X. **h** Atypical clear cell area, 400X. **i** Cystic area, 400X

**Fig. 3:**
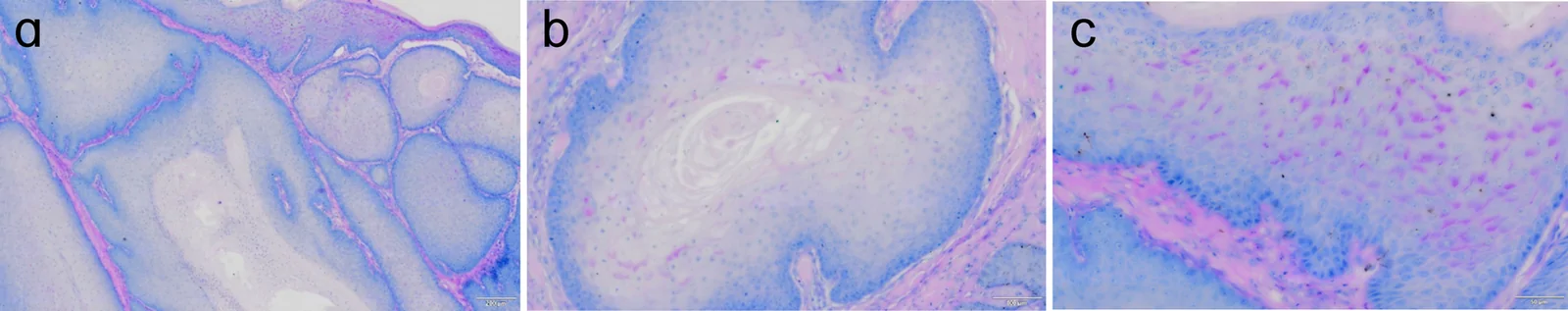
PAS staining of tumor tissue sections. **a** 40X. **b** 100X. **c** 200X. The Glycogen PAS Staining Kit (Beijing Solarbio Science & Technology Co., Ltd., Beijing, China) was used

**Fig. 4:**
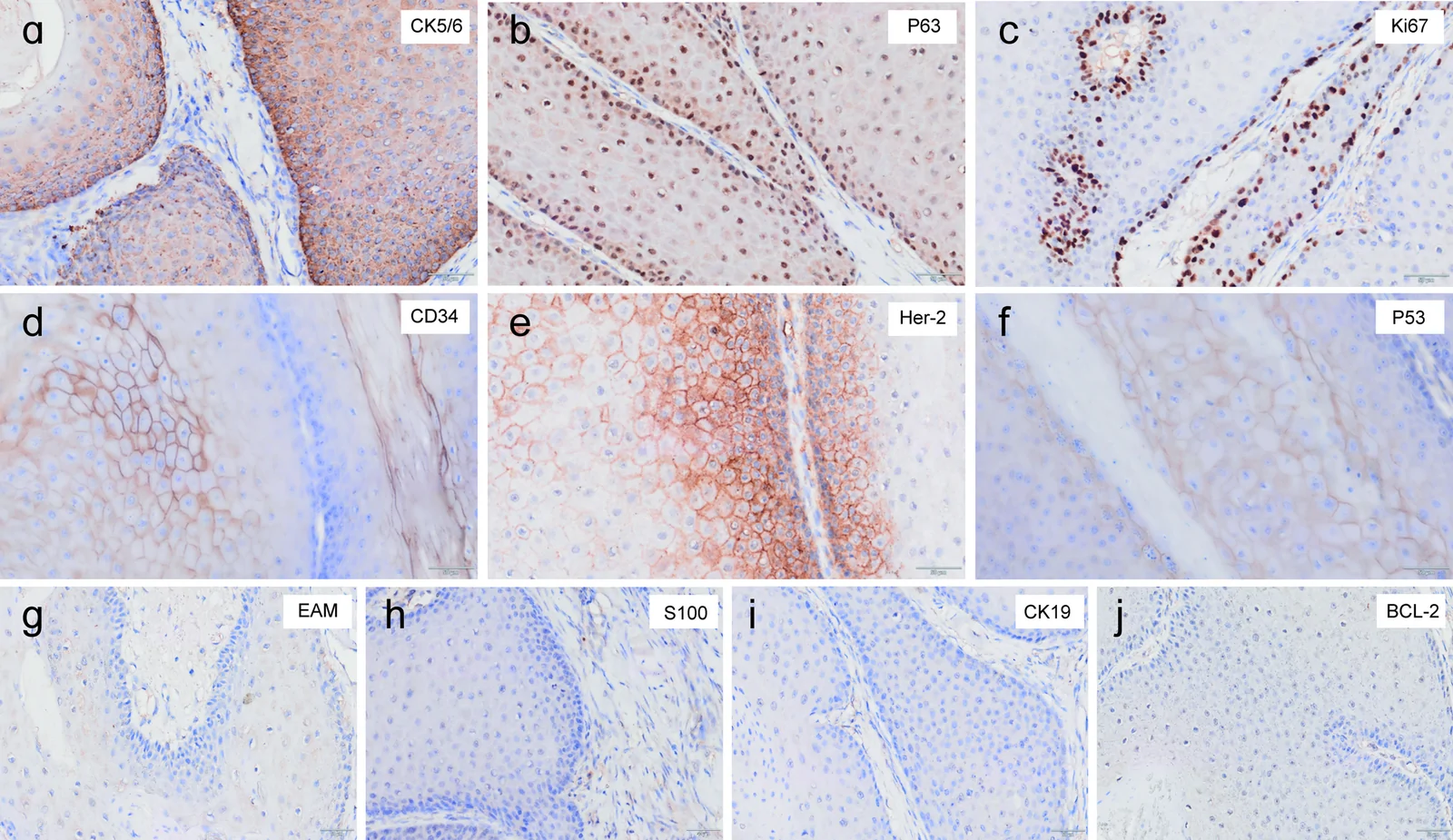
Immunohistochemistry of tumor tissue sections. **a **CK5/6 (++), 200X. **b** p63 (++), 200X. **c** Ki-67 (+), 200X. **d** CD34 (+), 200X. **e** Her-2 (+), 200X. **f** p53 (+), 200X. **g** EMA (-), 200X. **h** S-100 (-), 200X. **i** CK-19 (-), 200X. **j** BCL-2 (-), 200X. Immunohistochemistry was performed using the EnVision™ Detection Kit (Dako, Denmark). Primary antibodies included mouse anti‑human Bcl‑2, CK5/6, CK19, S100, EMA, CD34, p53 (all MXB Biotechnologies, China); p63 (ZSGB‑BIO, China); rabbit anti‑human Ki‑67 (MXB Biotechnologies, China) and Her‑2 (ROCHE, Switzerland)

Pathological observation revealed ulcers, telangiectasia, and superficial erosion on the skin surface. The tumor tissue was located in the dermis, with partial continuity to the epidermis, and exhibited lobular, cord-like, or nest-like growth patterns, with obvious invasive growth (Fig. 2a-c). In some areas, typical features of trichilemmal keratinization were observed: solid nests of squamous cells with peripheral palisading nuclei and abrupt central keratinization forming eosinophilic amorphous keratin material without a granular layer. Numerous typical and pathological mitoses were observed (Fig. 2d-e). In other regions, the tumor tissue was composed of atypical clear cells with disorganized arrangement, abundant glycogen, markedly pleomorphic and variable-sized nuclei, prominent nucleoli, and extremely rare mitotic figures (Fig. 2f-h). Some cystic areas were lined by outer root sheath-type epithelium. Solid cell nests were continuous with the cyst lining via precursor epithelial cells. The cytoplasm was clear resembling that of clear cells, with occasional mild cellular atypia and very rare mitoses (Fig. 2i). To confirm the diagnosis and exclude squamous cell carcinoma and trichofolliculoma, special staining and immunohistochemical (IHC) analyses were performed as follows. For special staining Periodic Acid-Schiff (PAS) staining showed positive reactions for glycogen and keratinous substances in tumor cells, and this positive reaction was more prominent in clear cells (Fig. 3). For immunohistochemical identification, positive expression was observed for Her-2 (+), CK5/6 (++), p63 (++), Ki-67 (+), CD34 (+), and p53 (+), while BCL-2, EMA, S-100, and CK-19 were negative (Fig. 4). Among these markers, CK5/6 (a high-molecular-weight keratin) and p63 (a follicular epithelial stem cell marker) confirmed the epithelial origin of the tumor and its association with hair follicle outer root sheath; CD34 positivity helped distinguish it from squamous cell carcinoma (which is usually CD34-negative); the presence of a large number of typical and pathological mitoses, combined with p53 positivity and focal Ki-67(+) expression, confirmed the malignant proliferative nature of the tumor; and Her-2 positivity was consistent with previous reports of ERBB2 gene amplification in human trichilemmal carcinoma, further supporting the diagnosis. Therefore, based on the above histomorphological findings together with the immunohistochemical results presented, the diagnosis of trichilemmal carcinoma was established.

### Discussion and conclusions

Previous studies have clearly indicated that trichilemmal carcinoma (TC) is a rare malignant tumor of cutaneous appendages in humans, with an extremely low incidence rate [7]. Furthermore, there are no reported cases or relevant research studies on this tumor in animals worldwide.

In this case, a 23-year-old male giant panda was diagnosed with TC following verification by histopathological examination and immunohistochemistry. This finding overturns the traditional perception that “trichilemmal carcinoma occurs exclusively in humans,” suggesting that the onset of this tumor may not be limited to a single species and that there may be cross-species commonalities in its evolutionary and pathogenic mechanisms.

From the biological perspective of the giant panda, this individual had entered the elderly stage, and its immune function might have naturally declined with aging. Meanwhile, although this giant panda had been in a controlled environment of artificial breeding, long-term exposure to environmental stimuli might still induce the malignant transformation of outer root sheath cells of hair follicles. This aligns closely with the pathogenic factors of “ultraviolet radiation, chronic inflammation, and low immune function” in human trichilemmal carcinoma [8], providing key clues for subsequent research on cross-species tumor mechanisms (such as genetic mutations and abnormal signaling pathways). TC in humans is mostly concentrated in hair-dense areas such as the scalp and face [9, 10]. Since these regions are rich in outer root sheath cells of hair follicles, they are more susceptible to stimulation by pathogenic factors. In this case, the tumor arose in the scrotal skin of a giant panda. Although this area is covered with hair, it is not considered a “high-incidence site” in traditional understanding. This discrepancy may be related to the anatomical and physiological characteristics of giant pandas: the scrotal skin is in a chronically moist environment, making it vulnerable to friction, sweat irritation, and microbial invasion, which may induce local chronic inflammation. Meanwhile, the scrotal skin differs from the scalp in terms of hair follicle density and cell proliferation activity, and the malignant transformation threshold of its outer root sheath cells may be lower. This finding suggests that the site selection for the occurrence of trichilemmal carcinoma may be influenced by species-specific anatomical structures and physiological environments. In the future, more animal cases need to be incorporated to clarify the “site-etiology” correlation pattern.

Neutrophils, GLB, ALP, AFU, and LDH were all elevated to varying degrees in this case. Neutrophilia is a typical bodily response to inflammation or tissue damage. In this case, there were clear ulcers, bleeding, and necrosis on the surface of the lesion, suggesting that the local area might be complicated by bacterial infection or tumor-associated inflammation. Inflammatory factors (such as IL-1β and TNF-α) released by tumor cells can stimulate the proliferation of neutrophils [11], while infection further exacerbates the inflammatory response, forming a “tumor-inflammation” vicious cycle. The elevation of globulin may be associated with increased synthesis of immune proteins induced by chronic inflammation, reflecting the body’s immune compensatory mechanism activated to combat tumors or infections [12].This is consistent with the patterns observed in human cancer patients. LDH is widely present in tissues throughout the body. Its elevation indicates cell damage or active cell proliferation. In this case, the invasive growth of the tumor may have caused necrosis of local tissues, leading to the release of LDH into the bloodstream [13].

Pathological observations revealed that the tumor infiltrated the dermal layer in a lobular and cord-like pattern, with some parts connected to the epidermis. The tumor cells contained abundant glycogen (positive for PAS staining) [14]; cells at the periphery of the lobules were arranged in a palisade pattern, and “abrupt keratinization without a granular layer” was observed in the center. These features are the core diagnostic criteria for human trichilemmal carcinoma, especially “outer root sheath keratinization” and “palisade arrangement”—which can effectively distinguish it from squamous cell carcinoma (no palisade arrangement, keratinization with a granular layer) and trichilemmoma (benign, no invasive growth) [15].

Immunohistochemistry showed positive expression of 6 proteins, including CK5/6, p63, CD34, Ki-67, p53, and Her-2. Among them, CK5/6 is a high-molecular-weight keratin that is specifically expressed in hair follicle epithelium [16]. p63 is a marker for hair follicle stem cells [17]. Together, they confirm that the tumor originates from the outer root sheath of the hair follicle and rule out non-epithelial tumors [18]. CD34 is mostly positive in human trichilemmal carcinoma, while it is mostly negative in squamous cell carcinoma. This marker serves as a key basis for distinguishing this case from squamous cell carcinoma [19]. Positive p53 indicates that tumor cells may have TP53 gene mutation (a common mutation in human trichilemmal carcinoma), and positive Ki-67 reflects the malignant proliferative activity of the tumor [20]. Together, they confirm the malignant nature of the tumor. Consistent with the research conclusion that “ERBB2 gene amplification leads to Her-2 overexpression” in human trichilemmal carcinoma [2], the positive Her-2 in this case further supports the cross-species molecular mechanism commonality of trichilemmal carcinoma.

The preferred treatments for human trichilemmal carcinoma are “wide local excision” or “Mohs micrographic surgery”, with the core goal of ensuring an adequate tumor-free margin and reducing the risk of local recurrence [21]. In this case, the veterinary team performed “wide local excision under general anesthesia”. This plan fully adhered to the treatment principles for human trichilemmal carcinoma, while optimizing the operation based on the giant panda’s body size and physiological characteristics. For postoperative wound repair, flap transplantation is often used to achieve tension-free suture and reduce scar formation.

This case provides the first animal sample for cross-species research on trichilemmal carcinoma. In the future, genetic sequencing (such as detection of TP53, ERBB2, and MOB1 mutations) can be used to clarify the molecular differences between giant panda and human tumors, and explore the interaction mechanism of “species-gene-environment”. Meanwhile, an animal model (e.g., MOB1 gene knockout mice) can be established based on this case to verify the function of pathogenic genes, thereby filling the gap in the mechanism research of trichilemmal carcinoma.

## Data Availability

All data generated or analyzed during this study are included in this published article.

## Abbreviations

TC: Trichilemmal Carcinoma
PAS: Periodic Acid-Schiff
Her-2(HER2, ERBB2): Human Epidermal growth factor Receptor 2
CK5/6: Cytokeratin
P63: Tumor protein 63
Ki-67: Kiel-67
CD34: Cluster of Differentiation 34
TP53(p53): Tumor Protein 53
H&E: Hematoxylin-Eosin
BRD4: Bromodomain protein 4
TYMS: Thymidylate Synthase
MOB1: MOB kinase activator 1
CEA: Carcinoembryonic Antigen
EMA: Epithelial Membrane Antigen
GLB: Globulin
GR: Glutathione reductase
IVC: Inferior Vena Cava
ALP: Alkaline phosphatase
AFU: Alpha-L-Fucosidase
LDH: Lactate dehydrogenase
IHC: Immunohistochemistry
BCL-2: B-cell lymphoma 2
S-100: S100 calcium binding protein
CK-19: Cytokeratin 19
IL-1β: Interleukin-1β
TNF-α: Tumor Necrosis Factor-alpha
